# Active regulatory T-cells contribute to broadened T-cell repertoire diversity in ivIg-treated SLE patients

**DOI:** 10.1186/1479-5876-9-S2-P6

**Published:** 2011-11-23

**Authors:** Nuno Costa, Ana E Pires, Ana M Gabriel, Luiz F Goulart, Clara Pereira, Barbara Leal, Ana C Queiros, Wahiba Chaara, Maria F Moraes-Fontes, Carlos Vasconcelos, Carlos Ferreira, Jorge Martins, Marina Bastos, Maria J Santos, Maria A Pereira, Berta Martins, Margarida Lima, Cristina João, Adrien Six, Jocelyne Demengeot, Constantin Fesel

**Affiliations:** 1 Instituto Gulbenkian de Ciência, Oeiras, Portugal; 2 IPO-CIPM/CEDOC-FCM, Lisboa, Portugal; 3 ICBAS, Porto, Portugal; 4 UPMC-Paris-6/CNRS-UMR7211/AP-HP, Paris, France; 5 Hospital Sto-António, Porto, Portugal; 6 Hospital Sta-Maria, Lisboa, Portugal; 7 Hospital Marmeleiros, Funchal, Portugal; 8 Hospital São-Teotónio, Viseu, Portugal; 9 Hospital Garcia de Orta, Almada, Portugal; 10 Hospital de Faro, Faro, Portugal

## 

Intravenous IgG (ivIg) is a therapeutic alternative for lupus erythematosus. Relative oligoclonality of circulating T-cells in SLE has been reported. Also CD4+Foxp3+ regulatory T-cells (Tregs) have a characteristically reduced activity in SLE, reflected by CD25 surface density. Aiming to study the role of Tregs for ivIg therapy, we characterized Tregs and determined TCR spectratypes of four Vb families with reported oligoclonality, in 15 lupus patients (14 with SLE and one with discoid LE) treated with ivIg in cycles of 2-6 consecutive monthly infusions. Among these 15 patients, 11 responded with clinical improvement. Cell counts, cytometry and TCR spectratypes were obtained from peripheral blood at various time points before, during and after ivIg treatment. T-cell oligoclonality was assessed as Vb-familywise repertoire perturbation, calculated for each patient in respect to an individual reference profile averaged over all available time points. For 11/15 patients, average Vb1/Vb2/Vb11/Vb14 repertoires were less perturbed under ivIg treatment than outside ivIg therapy. The four exceptions with relatively increased average perturbation during ivIg therapy included three patients who failed to respond clinically to an ivIg therapy cycle. Patients' Treg CD25 surface density (cytometric MFI) was, other than Treg/CD4+ frequency, clearly reduced when compared to healthy controls, but not obviously influenced by ivIg. However, patients' average Treg CD25 MFI was found negatively correlated with both Vb11 and Vb14 perturbations measured under ivIg therapy, which indicates a role of active Tregs in the therapeutic effect of ivIg.

